# Supplementary material for: A Novel G-Quadruplex Binding Protein in Yeast—Slx9
Source: Molecules. 2019 May 7;24(9):1774. doi: 10.3390/molecules24091774 (PMC6539110; doi:10.3390/molecules24091774)
Supplement: Supplementary file 1 [file molecules-24-01774-s001.pdf]

Supplementary online Material

Supplemental File S1: Yeast and bacteria strains used in this study.

Yeast strains used in this study.

**Strain**	**Genotype**	**Source**	**Experiment**
W303	*MATa ura3-1 trp1Δ 2 leu2-3, 112 his3-11,15 ade2-1 can1-100*	R. Rothstein	wild type
Y1H Gold	*MATα ura3-52 his3-200 ade2-101 trp1-901 leu2-3, 112 gal4Δ gal80Δ met- MEL1*	Clontech	Y1H
YPH500	*MATα ura3-52 lys2-801 ade2-101 trp1Δ63 his3Δ200 leu2Δ1*	[26]	GCR

Yeast strains created in this study.

**Name**	**Genotype**	**Created by**	**Experiment**
SG46	Y1HGold G4$_{IX}$	SG	Y1H
SG101	Y1HGold G4$_{mut}$	SG	
SG386	W303 *slx9::TRP1*	SG	Growth assay, spot assay
SG917	W303 *slx9::TRP1 sgs1::HIS3*	SG	
SG922	W303 *sgs1::HIS3*	SG	
KP77	W303 *pif1-m2*	[8]	Spot assay
SP2F4	W303 *slx9::TRP1 pif1-m2*	SP	
SG1010	W303 Slx9-Myc *TRP1*	SG	ChIP–qPCR
SP2F7	W303 *sgs1::HIS3* Slx9-Myc *TRP1*	SP	
			GCR
SG1023	YPH500 *sgs1::HIS3*	SG	
KW200	YPH500 *prb1::*control-sequence-*LEU2*	[28]	
SG1076	YPH500 *prb1::*control-sequence-*LEU2 sgs1::HIS3*	SG	
SG431	YPH500 *slx9::TRP1*	SG	
SG66	YPH500 *prb1::*G4$_{IV}$-*LEU2*	SG	
SG654	YPH500 *prb1::*G4$_{IV}$-*LEU2 slx9::TRP1*	SG	
KW203	YPH500 *prb1::*G-rich-*LEU2*	[28]	
SB17	YPH500 *prb1::*G-rich-*LEU2 slx9::TRP1*	SB	
SB21	YPH500 *prb1::*control sequence-*LEU2 slx9::TRP1*	SB	

Y1H screening strains created in this study. Shown is the bait sequence and the minimal inhibitory concentration of aureobasidin A, which was used in the screening and for the retransformations. G-tracts or C-tracts are in bold, mutations are marked red.

Strain	Bait sequence (5’-3’)	AbA [ng/ml]
SG46 *bait*-G4	G4 chromosome IX ($G4_{IX}$):<br>TCCGAAATTTTGATTTGGAGACTGATTTGGA**GGG**TACGGT**GGG**TAATAA**GG**<br>**G**AAGGTATC**GGG**ATTGGGGTAGGCCATTAAGGGATAATTCCATTGCCATTG	100
SG101 *bait*-mut-G4	Mutated G4 chromosome IX (mut-$G4_{IX}$):<br>TCCGAAATTTTGATTTGGAGACTGATTTGCA**GCG**TACGGT**GCG**TAATAA**CG**<br>**C**AAGCTATC**GCG**ATTGCCGTAGCCCATTAAGGGATAATTCCATTGCCATTG	400

E. coli strains used in this study.

Strain	Genotype	Source	Experiment
DH5α	*F^- ϕ80dlacZΔM15 endA1 recA1 hsdR17(r_K^-,m_K^+) supE44 thi-1 gyrA96(Nal^r) relA1 Δ(lacZYA-argF)U169 λ^-*	[64]	Cloning
XL1-Blue	*endA1 gyrA96(nal^R) thi-1 recA1 relA1 lac glnV44 F'[::Tn10 $proAB^+$ $lacI^q$ Δ(lacZ)M15] hsdR17(r_K^- m_K^+)*	Stratagene	Y1H
Rosetta pLysS	*F^- ompT hsdS*$_B$(r_B^- m_B^-) *gal dcm* (DE3) pLysSRARE (Cam^R)	Novagen	Slx9 purification

E. coli strains created in this study.

Name	Genotype	Created by	Experiment
SG22	DH5α pAbAi-$G4_{IX}$	SG	Y1H
SG70	DH5α pAbAi-$G4_{mut}$	SG	Y1H
SG45	DH5α pRS415-$G4_{I}$	SG	GCR
SG43	DH5α pRS415-$G4_{IV}$	SG	GCR
SG146	Rosetta pLysS pET28a-*SLX9*	SG	Slx9 purification

Supplemental File S2: List of G4 folding oligodeoxynucleotides.

Sequences of oligonucleotides used for *in vitro* binding studies. Mutations are marked red, G-tracts of G4 motifs are printed in bold.

Name	Sequence 5’-3’	Use
$G4_{IX}$	AAAAAAAAAA**GGG**TACGGT**GGG**TAATAA**GGG**AAGGTATC**GGG**	G4-folding *in vitro*
$G4_{mut}$	AAAAAAAAAAGCGTACGGTGCGTAATAACGCAAGCTATCGCG	
$G4_{TP}$	AAAAAAAAAA**GGGGG**AGCT**GGGG**TAGAT**GGG**AATGTGA**GGG**	
$G4_{rDNA}$	AAAAAAAAAA**GGG**TAAC**GGGG**AATAA**GGG**TTCGATTCCGGAGA**GGG**	
dsDNA	GACGCTGCCGAATTCTGGCTTGCTAGGACATCTTTGCCCACGTTGACCCG + CGGGTCAACGTGGGCAAAGATGTCCTAGCAAGCCAGAATTCGGCAGCGTC	Control DNA structures for *in vitro* binding studies. Sequences from [35]
bubble	CGGGTCAACGTGGGCAAAGCCAATGCGATCGGCCAGAATTCGGCAGCGTC + GACGCTGCCGAATTCTGGCTTGCTCGGACATCTTTGCCCACGTTGACCCG	

fork	GACGCTGCCGAATTCTGGCTTGCTAGGACATCTTTGCCCACGTTGACCCG + CGGGTCAACGTGGGCAAAGATGTCCTAGCAATGTAATCGTCTATGACGTC	
4-fork	GACGCTGCCGAATTCTGGCTTGCTAGGACATCTTTGCCCACGTTGACCCG + CGGGTCAACGTGGGCAAAGATGTCCTAGCAATGTAATCGTCTATGACGTC + GACGTCATAGACGATTACATTGCTAGGACATGCTGTCTAGAGACTATCGC + GCGATAGTCTCTAGACAGCATGTCCTAGCAAGCCAGAATTCGGCAGCGTC	

Supplemental File S3: List of peaks of ChIP-seq.

Supplemental File S4: List of primers used in this study.

Sequences of oligodeoxynucleotides used for conventional ChIP–qPCR experiments.

No.	Sequence 5’-3’	Orientation	Feature
1	ATAAAAGGTAGAAAGAATT	fw	Non-G-rich 1
	CGGTTTGGCTTGTATTGCC	rev	
2	ATATCAATACATCACACCTG	fw	Non-G-rich 2
	GGAAAAAAGAGCAGCAATAC	rev	
3	GCTGTCTGGTTAGTCTTCG	fw	G-rich 1
	CTTCGTACAGACCTTCACCC	rev	
4	CCACCTACAGATTATAGAAG	fw	G-rich 2
	CAAGGTGTATGACGTGCCG	rev	
5	GCTTCAGCCTGGGGTAAC	fw	XIIIb: G4 (Tract3), G4 1
	GGCACCATTAGATTCACCAC	rev	
6	AATCCCGTCGCTATGCTC	fw	XI: G4 (Tract3), G4 2
	CTCCCGGTCTGTTATTTTC	rev	
7	ATACGCAGTATGGTGATATC	fw	XV (Tract3) G4 3
	GTTTATTGCCGATATACCTC	rev	
8	CCCTTATCAAAGCAAATGCG	fw	Control 1
	GACCACTTGATTGACTAGATC	rev	
9	CGATTATTAGTAGCGCAAAG	fw	Control 2
	CTTGTTATATCAATAACATG	rev	

Sequences of oligodeoxynucleotides for expression quantification by qPCR.

Sequence 5’-3’	Orientation	Use
TACAGTGCATGTGGTATGAC	fw	Expression *RTC2*
GAGCTATACGATTGGCTATC	rev	
ATCGCTAGATACTCCAATGG	fw	Expression *SKP2*
TAAGTGCAGTGATAGGAGAC	rev	
GTATCCAGGACTCATTCAAC	fw	Expression *PRB1*
TAGCCTCGACAATAGAGTC	rev	
GAAAGGCGAACTACGAGTG	fw	Expression *PCD1*

GACCTCAGGATCATGCGG	rev	
AATGGACAGTCCACGAAGC	fw	Expression *TMA10*
TCTCATCGCCAGGCTTGC	rev	
CCTAGCAAGAGTTGACTGC	fw	Expression *YPK2*
TAGACTGAATCGTCCCTCG	rev	
CCGAACGTAGGAGTAGCG	fw	Expression *IGD1*
GTCTGTTGCCAGCACGAG	rev	
CTGTTGTACGGCAATATCGA	fw	Expression *YLR312C*
TCCAGCCCATTAGGTCATC	rev	
TAGGAGCAGCCCTGTACG	fw	Expression *RRT6*
TCTTGCAACACGTCCATTAC	rev	
CAGTCAAACTTCCTATCGAG	fw	Expression *MDH2*
ACAACACACTTATAACCGGC	rev	
CGCTCCTCGTGCTGTCTTCC	fw	Expression Actin
CAGGGTGTTCTTCTGGGGCAAC	rev	
CAGACATCAGCGTACTAGG	fw	Expression *UTP6*
TGGCATAGTAACCCTTATGC	rev	

Supplemental File S5: Supporting information for binding analysis.

Supplemental File 5, Götz et al., 2019

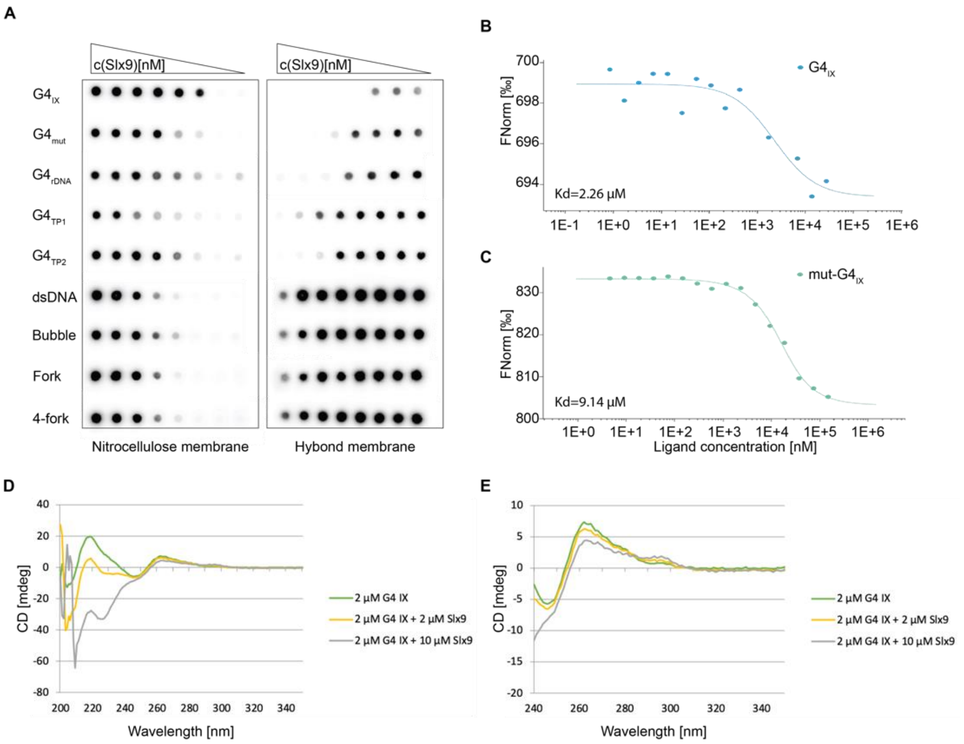

(A) Dotblot. (B) MST: Binding reactions were prepared in PBS supplemented with 400 mM NaCl, 0.5% BSA, and 0.05% Tween-20 in a total volume of 40 µL. For binding reactions 25 nM 5´Cy5-labeled oligonucleotides (folded G4s and controls) were used (see Supplementary Table S2. Different concentrations of Slx9 ranging from 27.5 µM to 0.03 nM and a constant concentration of DNA (25 nM) were used. (B,C) MST analysis was performed using standard capillaries from Nanotemper with LED 40%, 40% MST power, on the Monolith NT.115 instrument temperature 24 °C. (B) Slx9 binding to a G4 structure from chromosome IX was tested. (C) Slx9 binding to mutated G4 from chromosome IX. K_d was calculated using the MO Affinity analysis software.

(D,E) CD spectra of folded oligonucleotides $G4_{IX}$ (green) plus the addition of 2 µM Slx9 and 10 µM Slx9, respectively. G4 structures show the distinct minimum (243 nm) and maximum (264 nm) peaks indicating the presence of a parallel G4. Upon Slx9 titration changes in

ellipticity between 200 and 300 nm are observed. (E) Zoom of second maxima peak in D. A minor decrease of the G4 peak (green) after Slx9 titration is detected indicating that no or only minor changes on G4 stability occur due to Slx9 binding. CD spectra were recorded on a Jasco J-810 spectropolarimeter at 20°C with parameters as described previously [28].

Supplemental File S6: Slx9 has no G4-mediated effect on genome stability or transcriptional changes.

Supplemental File 6, Götz et al., 2019

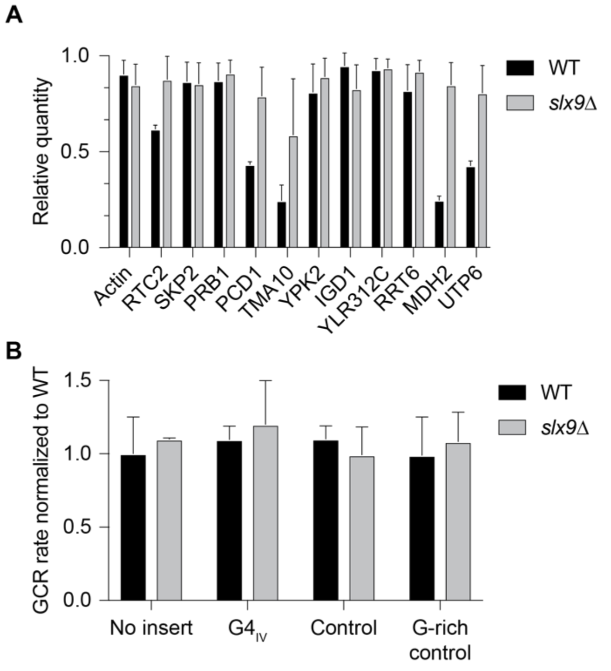

(A) Analysis of gene expression changes in *slx9Δ* strains (grey) compared to WT (black). (B) GCR analysis of *slx9Δ* strains. *slx9Δ* does not influence genome stability. Shown is the change of the GCR rate of the strain compared to wild type without insert at the *PRB1* locus ± standard error. Biological replicates n = 3. Sequences (5'–3') of the inserts at the *PRB1* locus in the GCR strains. Only the relevant part of the insert is depicted.

$G4_{IV}$: GGGGAGGGGAAGGGGAGGGG,

Control:

CTAATCTTTCAGCGTTGTAAATGTTGGTACCCAAACCCAATTGTCTACAAGTTTCCTTAGC,

G-rich control:

ATGGTGGTCATCTCAGTAGATGTAGAGGTGAAAGTACCGGTCCATGGCTCGGT.

Supplemental Methods

Gross chromosomal rearrangement assay

The gross chromosomal rearrangement (GCR) assay was performed as published with minor modifications [54]. Seven colonies per strain were grown for 48 h and cells were plated on different media: YPD (reference plate) and 5-FOA/Canavanine (selective plate). After incubation, the colonies were counted and the GCR rate was determined by fluctuation analysis using FALCOR and the MSS maximum likelihood method [56].

RNA isolation

RNA was isolated mainly according to the manufacturer's protocol. Briefly, cells were grown to an OD_{660} of 0.8 and RNA was isolated using the "High Pure RNA Isolation Kit" (Roche). 10^8 cells in log phase were harvested and lysed with glass beads for 1 min in a FastPrep-24 (MP). RNA was quantified using a spectrophotometer and its quality was assessed on an agarose gel. cDNA was synthesized from 5 µg RNA using Superscript II Reverse Transcriptase (Invitrogen) and oligo(dT) as per the manufacturer's instructions. qPCR was performed with SsoAdvanced SYBR Green Supermix (Bio-Rad) using the primers mentioned in Supplemental File S4.
